# A Novel Plasticization Mechanism in Poly(Lactic Acid)/PolyEthyleneGlycol Blends: From T_g_ Depression to a Structured Melt State

**DOI:** 10.3390/polym18030317

**Published:** 2026-01-24

**Authors:** Nawel Mechernene, Lina Benkraled, Assia Zennaki, Khadidja Arabeche, Abdelkader Berrayah, Lahcene Mechernene, Amina Bouriche, Sid Ahmed Benabdellah, Zohra Bouberka, Ana Barrera, Ulrich Maschke

**Affiliations:** 1 Laboratoire de Recherche sur les Macromolécules (LRM), Faculté des Sciences, Université Abou Bekr Belkaid de Tlemcen, BP 119, Tlemcen 13000, Algeria; 2 Unité Matériaux et Transformations (UMET), UMR 8207, Université de Lille, CNRS, INRAE, Centrale Lille, 59000 Lille, France; 3 Laboratoire Physico-Chimie des Matériaux-Catalyse et Environnement (LPCMCE), Université des Sciences et de la Technologie d’Oran Mohamed Boudiaf (USTOMB), BP 1505, El M’naouer, Oran 31000, Algeria

**Keywords:** polylactic acid (PLA), plasticization, poly(ethylene glycol) (PEG), thermorheology, miscibility, crystallinity, dynamic mechanical analysis (DMA), transient network, biodegradable polymers

## Abstract

Polylactic acid (PLA) is a promising biodegradable polymer whose widespread application is hindered by inherent brittleness. Polyethylene glycol (PEG) is a common plasticizer, but the effects of intermediate molecular weights, such as 4000 g/mol, on the coupled thermal, mechanical, and rheological properties of PLA remain insufficiently understood. This study presents a comprehensive analysis of PLA plasticized with 0–20 wt% PEG 4000, employing differential scanning calorimetry (DSC), dynamic mechanical analysis (DMA), and rheology. DSC confirmed excellent miscibility and a significant glass transition temperature (T_g_) depression exceeding 19 °C for the highest concentration. A complex, non-monotonic evolution of crystallinity was observed, associated with the formation of different crystalline forms (α′ and α). Critically, DMA revealed that the material’s thermo-mechanical response is dominated by its thermal history: while the plasticizing effect is masked in highly crystalline, as-cast films, it is unequivocally demonstrated in quenched amorphous samples. The core finding emerges from a targeted rheological investigation. An anomalous increase in melt viscosity and elasticity at intermediate PEG concentrations (5–15 wt%), observed at 180 °C, was systematically shown to vanish at 190 °C and in amorphous samples. This proves that the anomaly stems from residual crystalline domains (α′ precursors) persisting near the melting point, not from a transient molecular network. These results establish that PEG 4000 is a highly effective PLA plasticizer whose impact is profoundly mediated by processing-induced crystallinity. This work provides essential guidelines for tailoring PLA properties by controlling thermal history to optimize flexibility and processability for advanced applications, specifically in melt-processing for flexible packaging.

## 1. Introduction

Polylactic acid (PLA) is a biodegradable polymer characterized by attractive properties including thermoplasticity, high strength, and transparency [1,2,3]. However, its rigidity, relatively low strain at break, and thermal instability limit its use in applications such as food packaging [4,5]. To overcome these drawbacks, plasticization has been widely employed as an effective strategy. Plasticizers enhance PLA’s flexibility and processability by facilitating chain mobility [6,7,8,9,10], reducing hardness, density, viscosity, and electrostatic charge while increasing chain flexibility, breaking strength, and dielectric constant [11]. Other properties are also affected, such as the degree of crystallinity and resistance to biological degradation [11,12].

Among commonly used plasticizers such as polyethylene glycol (PEG), ethylene carbonate (EC), and propylene carbonate (PC) [7,8], PEG is particularly valued for its biodegradability, nontoxicity, and miscibility with PLA across a broad molecular weight range [7]. Studies by Li et al. [9] and Pillin et al. [10] have confirmed that the addition of PEG significantly improves the ductility and flexibility of PLA. The influence of PEG on PLA properties has been extensively investigated [13,14,15]. PEG’s miscibility with PLA stems from similar solubility parameters and specific interactions between terminal hydroxyl groups of PEG and carboxyl groups in PLA, as described by the Flory–Huggins theory. Molecular dynamics simulations by Takhulee et al. [16] indicate a low interaction parameter, confirming miscibility at low PEG concentrations (10–30 wt%), with phase separation predicted at higher concentrations.

This miscibility is strongly influenced by PEG molecular weight and content [9,13,15,17,18,19]. For example, Benkraled et al. [20] demonstrated that low-molecular-weight PEG (M_n_ = 400 g/mol) efficiently reduces the glass transition temperature (T_g_) and the cold crystallization temperature (T_cc_), while increasing crystallinity and crystallization rate. Similarly, Baiardo et al. [17] reported that low-molecular-weight PEG exhibits superior miscibility and plasticizing efficiency. In contrast, Li et al. [9] found that higher-molecular-weight PEG (M_n_ = 10,000 g/mol) improves crystallization capacity and impact toughness but may lead to phase separation at higher concentrations. Several studies also note that PEG accelerates spherulitic growth and raises the degree of crystallinity in PLA [9,18,19,21,22], with Greco et al. [19] highlighting a notable increase in crystallization rate. The ongoing relevance of this system is underscored by recent applications. For instance, Shin et al. [23] developed ductile PLA/PEG blend films for eco-friendly flexible packaging, noting significant improvements in crystallization and elongation at break. Furthermore, the utility of PLA/PEG extends into advanced fields like additive manufacturing and tissue engineering, where it is used to create high-performance 3D printing filaments [24] and structured scaffolds for bone and cartilage regeneration [25,26].

Nevertheless, important aspects remain underexplored. For example, the glass transition temperature T_g_ of pure PEG—which varies markedly with molecular weight and often lies at low temperatures—has rarely been directly measured and usually requires specialized equipment [27]. Faucher et al. [27] reported an unusual molecular weight dependence of T_g_ in poly(ethylene oxide), ranging from −95 °C for ethylene glycol to a maximum of −17 °C at M_n_ ≈ 6000 g/mol, followed by a decrease to −53 °C for very high molecular weights. This behavior, which is attributed to crystallinity effects in intermediate molecular weights, is often overlooked, and the T_g_ of PEG in PLA blends is frequently estimated using the Fox equation [28] rather than being measured directly.

Moreover, systematic comparisons of T_g_ and crystallinity (χ_c_) across different molecular weights and processing methods are scarce. Martin et al. [2] reported for PEG1500 (10 and 20 wt%) T_g_ values of 41 °C and 30 °C and χ_c_ values of 17% and 25%, respectively, while for PEG400, T_g_ dropped to 30 °C and 12 °C with χ_c_ reaching 26% and 29%. Benkraled et al. [20] observed similar values for PEG400 (T_g_ ≈ 32 °C and 16.5 °C at 10 and 20 wt%, respectively), despite using a different processing route (casting vs. melt blending). These results suggest that both molecular weight and processing history influence T_g_ and χ_c_, yet a comparative analysis of PEG with intermediate molecular weights—such as PEG4000—is largely missing. This gap is notable, as PEG4000 sits within the molecular weight range where Faucher et al. [27] reported unique T_g_ behavior for neat PEG, prompting questions about its effect within a PLA blend.

The crystallization behavior of PLA further complicates this picture, as PLA can crystallize into α, β, and γ forms depending on crystallization conditions [21,22,29]. Research indicates that PLA typically develops α and α′ forms, with their formation being influenced by crystallization temperature and PLA molecular weight [7,30,31,32]. The relationship between plasticization, crystalline form development, and final properties remains an active area of investigation, as optimizing this balance is key to advancing PLA from packaging to high-performance technical applications [33,34,35,36].

In this context, the present work focuses on the plasticization of PLA using PEG with a molecular weight of M_n_ = 4000 g/mol, a range that has received limited attention. This study thoroughly examines the effect of PEG4000 on the thermal, dynamic mechanical, and rheological properties of PLA/PEG blends, with particular emphasis on miscibility limits, T_g_ behavior, and crystallinity development. Special attention is given to the evolution of the crystalline morphology, with DSC analysis suggesting the possible presence of α and α′ crystalline forms and their correlation with enhanced crystallization. A key novelty of this work lies in the direct comparison with earlier studies: Benkraled et al. [20] (PEG400, casting) and Martin et al. [2] (PEG400 and PEG1500, melt blending). This approach allows us to decouple the effects of PEG molecular weight and processing method, providing new insights into the structure–property relationships in PLA/PEG blends. The miscibility and performance of the blends are evaluated experimentally using DSC, DMA, and dynamic rheometry, supported by theoretical analysis.

Through this comprehensive investigation, we aim to clarify the role of intermediate molecular weight PEG in tailoring PLA properties and establish clearer guidelines for designing PLA-based materials with optimized performance.

## 2. Materials and Methods

### 2.1. Materials

Poly(lactic acid) (PLA), grade Ingeo™ Biopolymer 4043D, was supplied by NatureWorks LLC (Minnetonka, MN, USA). This thermoplastic resin, with a molecular weight of 96,000 g/mol, is commonly used in packaging films, cardstock, and graphic arts. The material has a density of 1.24 g/cm^3^, a melt flow index of 2–10 g/10 min (190 °C, 2.16 kg), a melting temperature range of 160–170 °C, and a glass transition temperature between 55–60 °C. The melt enthalpy of 100% crystalline PLA was taken as ΔH_f_^0^ = 93.1 J/g. Polyethylene glycol (PEG), with an average molecular weight of 4000 g/mol and a density of 1.24 g/cm^3^, was purchased from Merck (Darmstadt, Germany) and used as a plasticizer without further purification. Chloroform (commercial grade, Honeywell, Seelze, Germany) was used as the solvent for sample preparation.

### 2.2. Sample Preparation

PLA/PEG films with PEG contents of 1, 5, 10, 15, and 20 wt% were prepared via solvent casting. To minimize hydrolytic degradation, PLA pellets were first dried in a vacuum oven (Memmert, Schwabach, Germany) at 50 °C for 24 h. PEG was used as received. Both components were dissolved separately in chloroform using an orbital shaker (IKA, Staufen, Germany; 300–400 rpm, ambient conditions). The solutions were then combined and stirred for 3 h to ensure homogeneity, then cast into glass Petri dishes and left for three days at ambient temperature to allow solvent evaporation. The resulting films were further dried under vacuum at 40 °C for 24 h to remove any residual solvent. Neat PLA films were also prepared following the same procedure as a reference. The final film thickness was approximately 0.2 mm.

### 2.3. Characterization

#### 2.3.1. Differential Scanning Calorimetry (DSC)

Thermal analysis was performed using a TA Instruments Q2000 DSC (New Castle, DE, USA) equipped with an RCS90 cooling system. Temperature and heat flow calibration were carried out using high-purity indium. Samples (10–12 mg) were sealed in Tzero aluminum pans and tested under a nitrogen atmosphere (50 mL/min). A two-cycle heating protocol was applied:

- First cycle: Heating from 0 °C to 180 °C at 10 °C/min to erase thermal and mechanical history.
- Second cycle: Cooling from 180 °C to 0 °C at 10 °C/min, followed by reheating from 0 °C to 180 °C at the same rate. Isothermal holds of 2 min were applied at 0 °C and 180 °C in each cycle. The glass transition temperature (T_g_), melting temperature (T_m_), melting enthalpy (ΔH_m_), and cold crystallization temperature (T_cc_) were determined from the second heating scan.

#### 2.3.2. Dynamic Mechanical Analysis (DMA)

Dynamic mechanical analysis was performed using a TA Instruments Q800 analyzer (New Castle, DE, USA) in tension mode. Rectangular samples (approximately 25 × 5 × 0.25 mm^3^) were prepared by hot-pressing solvent-cast films at 190 °C, followed by rapid quenching in ice water (~2 °C) to eliminate solvent history. All samples were stored in a desiccator at ambient temperature prior to measurement.

The DMA tests were conducted under the following conditions: an oscillation amplitude of 15 µm, a preload force of 0.01 N, and a frequency of 1 Hz. The temperature was ramped from room temperature to 180 °C at a heating rate of 3 °C/min. The storage modulus (E’), loss modulus (E’), and loss factor (tan δ) were recorded as functions of temperature.

The dynamic mechanical response of virgin samples (pure PLA and PLA/PEG blends) with their initial thermal history (i.e., without a prior heating-quenching cycle) was first characterized. These DMA results are compared directly with the data from the first heating cycle in DSC to correlate the viscoelastic transitions with the thermal behavior.

#### 2.3.3. Dynamic Rheological Measurements

The viscoelastic properties of the materials were characterized using small-amplitude oscillatory shear (SAOS) measurements. When a sinusoidal strain γ*$\left(t\right)=\gamma$_0_$e^{iwt}$ is applied to a linear viscoelastic material, the resulting stress response is σ*=σ_0_$e^{i(wt+\delta )}$, where γ_0_ and σ_0_ represent the strain and stress amplitudes, respectively, ω is the angular frequency, and δ is the phase shift between stress and strain.

The complex modulus G(ω) is defined as:

G(ω) = σ*/γ* = G′(ω) + iG″(ω) (1)

where

G′(ω) = (σ_0_/γ_0_) cos δ represents the storage modulus (2)

G″(ω) = (σ_0_/γ_0_) sin δ represents the loss modulus (3)

tan δ = G″(ω)/G′(ω) defines the damping factor(4)

The complex viscosity is derived as:

η(ω) = σ/γ˙* = G*(ω)/iω = η′(ω) − iη″(ω)(5)

with:

η′(ω) = G″(ω)/ω and η″(ω) = G′(ω)/ω(6)

and its magnitude given by:

(7) $$\left|\eta^{*}\left(\omega \right)\right|=\sqrt{{{ƞ}^{\prime }\left(\omega \right)}^{2}+{{ƞ}^{\prime \prime }(\omega )}^{2}}$$

To model the viscoelastic response, the Carreau–Yasuda equation, originally developed for steady-state viscosity η(γ˙), was applied to dynamic data via the Cox-Merz rule (η(γ˙) ≈ ${|\eta }^{*}\left(\omega \right)|$ for γ˙= ω) [37,38]:

(8) $${|\eta }^{*}\left(\omega \right)|=\eta_{0}^{*}{\left[1+{\left(\lambda \omega \right)}^{a}\right]}^{\frac{n-1}{a}}$$

where η_0_* is the zero-shear complex viscosity, λ represents the relaxation time, a is the Yasuda parameter indicating the transition width between Newtonian and shear-thinning regions, and n is the power-law index. For materials exhibiting a yield stress, the model was further modified as proposed by Lertwimolnun et al. [39] and Berzin et al. [40]:

(9) $${|\eta }^{*}\left(\omega \right)|=\frac{\sigma_{0}}{\omega }+\eta_{0}^{*}{\left[1+{\left(\lambda \omega \right)}^{a}\right]}^{\frac{n-1}{a}}$$

where σ_0_ represents the yield stress.

Sample Preparation and Experimental Protocol:

Rheological samples were prepared using the same protocol established for DMA to ensure consistency in thermal history. Solvent-cast films were hot-pressed at 190 °C and subsequently rapidly quenched in ice water to obtain a unified, predominantly amorphous initial state. This step is crucial in order to eliminate the confounding effects of solvent history and uncontrolled crystallinity, thereby allowing the intrinsic effect of PEG plasticization on melt rheology to be assessed.

Experimental measurements were performed using a Discovery Hybrid Rheometer-2 (TA Instruments, New Castle, DE, USA) which was equipped with 25 mm parallel plate geometry. To investigate the effect of temperature and potential residual crystallinity on the viscoelastic response—particularly in light of the double-melting behavior observed by DSC—frequency sweep tests were conducted at two distinct temperatures: 180 °C and 190 °C. For each test, the sample was equilibrated for 5 min at the target temperature. Frequency sweeps were then performed from 0.1 to 600 rad s^−1^ at a constant strain of 10%, confirmed to be within the linear viscoelastic regime. This comparative approach allows for assessing whether rheological anomalies are suppressed at higher temperatures, which would indicate a dominant role of residual crystalline structures.

This dual approach enables the discrimination between rheological anomalies arising from persistent residual crystallinity and those indicative of unique molecular-level interactions or transient network formation in the PLA/PEG blends.

## 3. Results and Discussion

### 3.1. Thermal Behavior of PLA/PEG Blends

**Figure 1:**
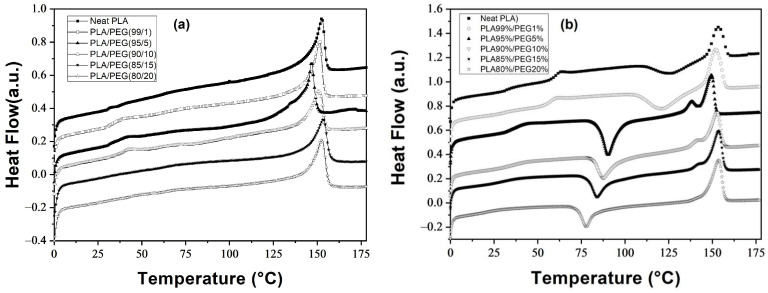
Differential scanning calorimetry (DSC) thermograms of neat PLA and PLA/PEG blends: (**a**) first heating scan, representing the initial thermal history; (**b**) second heating scan, obtained after erasing the thermal history.

The thermal properties of neat PLA and PLA/PEG blends were investigated using DSC. Figure 1a,b display the thermograms of the first and second heating scans, respectively. Neat PLA and blends with 1, 5, 10, 15, and 20 wt.% PEG were analyzed. Key parameters such as the glass transition temperature (T_g_), cold crystallization temperature (T_cc_), and crystallinity ratio (χ_c_) were evaluated, with data summarized in Table 1 and Table 2.

During the first heating scan (Figure 1a), pure PLA and all PLA/PEG blends exhibited a single melting peak near 150 °C and no cold crystallization peak (T_cc_). The absence of a detectable T_g_, lack of crystallization exotherms, and presence of a single melting peak confirmed that the samples were fully crystallized prior to analysis [21,22,27,28,29,30,31].

In the second heating scan (Figure 1b), after erasing thermal history, all samples exhibited distinct T_g_, T_cc_, and melting temperatures (T_m1_ and T_m2_). The thermograms revealed cold crystallization of PLA above T_g_, followed by melting between 130–160 °C. Blends with 5–15 wt.% PEG showed double melting peaks, which can be attributed to the melting-recrystallization mechanism, potentially involving the less ordered α’-form and the more stable α-form crystals [22,29,30,31,32,33,34]. At 20 wt.% PEG, only a single melting peak was observed, suggesting a predominance of the α-form under these conditions.

The crystallinity degree (χ_c_) was calculated using Equation (10)

(10) $$\chi_{c}=\frac{{∆H}_{m}}{W_{PLA}.{∆H}_{m}^{0}}\times 100$$

where $W_{PLA}$ is the weight fraction of PLA in the blend and ${∆H}_{m}^{0}$ = 93.1 J/g represents the melting enthalpy of fully crystalline PLA [41].

### 3.2. Miscibility and Fox Equation Analysis

The incorporation of PEG significantly reduced the glass transition temperature of PLA. The T_g_ decreased from 59.7 °C for neat PLA to 19.2 °C for the PLA/PEG 80/20 blend, representing a 40.5 °C reduction. This depression of T_g_ is a classic indicator of the plasticizing effect of PEG, which enhances the chain mobility of the PLA matrix [4,13,14].

A similar trend was observed for the cold crystallization temperature (T_cc_). The T_cc_ peak of neat PLA was observed at 124.3 °C, while the peaks for the plasticized samples became sharper and shifted to lower temperatures, reaching 77.8 °C for the PLA/PEG 80/20 blend (a decrease of 46.5 °C). This reduction in T_cc_ further supports the role of PEG in enhancing PLA’s chain mobility and improving its crystallization capacity. The concurrent decreases in both T_g_ and T_cc_ provide strong evidence for the miscibility of the PLA/PEG system at PEG concentrations up to 20 wt%.

For highly crystalline polymers or substances with ultralow T_g_ values, direct measurement may be impossible. However, blending such a material with an ultralow T_g_ with a compatible polymer allows its T_g_ to be determined using established models. The miscibility of the PLA/PEG blends was assessed using the Fox equation (Equation (11)) [28]:

(11) $$\frac{1}{T_{g}}=\frac{w_{1}}{T_{g1}}+\frac{w_{2}}{T_{g2}}$$

In this equation, T_g_ is the glass transition of the blend, while T_g1_ and T_g2_ are the T_g_ values of the components, and $w_{1}$ and $w_{2}$ are their weight fractions. Since the T_g2_ of PEG could not be determined directly from our DSC experiments, the Fox equation was rearranged into a linear form (Equation (12)):

(12) $$\frac{1}{T_{g}}=\left(\frac{1}{T_{g2}}-\frac{1}{T_{g1}}\right)w_{2}+\frac{1}{T_{g1}}$$

**Figure 2:**
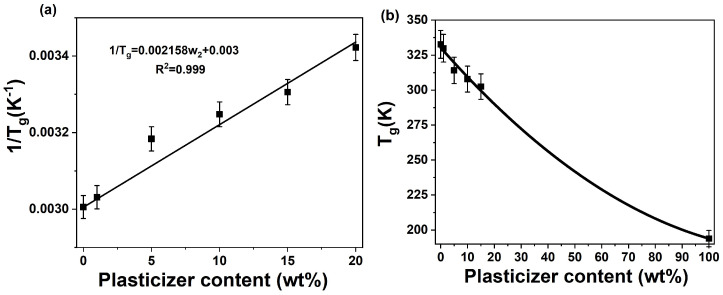
Glass transition temperature (T_g_) of PLA/PEG blends. (**a**) Linear relationship between the inverse T_g_ (1/T_g_) and PEG content (w_2_), with the solid line showing the linear fit. (**b**) Depression of T_g_ with increasing PEG content (w_2_): experimental data (symbols) and the prediction based on the Fox equation (solid line).

This linearized relationship indicates that miscibility is confirmed if the reciprocal of the measured T_g_ (1/T_g_) varies linearly with the plasticizer content ($w_{2}$). As shown in Figure 2a, an excellent linear correlation (R^2^ = 0.999) was found, confirming the miscibility of PLA and PEG within the 0–20 wt% concentration range. Furthermore, the T_g_ of PEG was estimated from the slope of the linear regression to be −79.1 °C, a value consistent with those reported in the literature.

This analysis demonstrates the efficacy of PEG 4000 as a plasticizer for PLA, reducing T_g_ and enhancing chain mobility while maintaining miscibility up to 20 wt.%. The Fox model reliably predicts blend behavior and provide insights for tailoring PLA properties in applications requiring low-temperature flexibility.

### 3.3. Crystallinity Evolution

**Figure 3:**
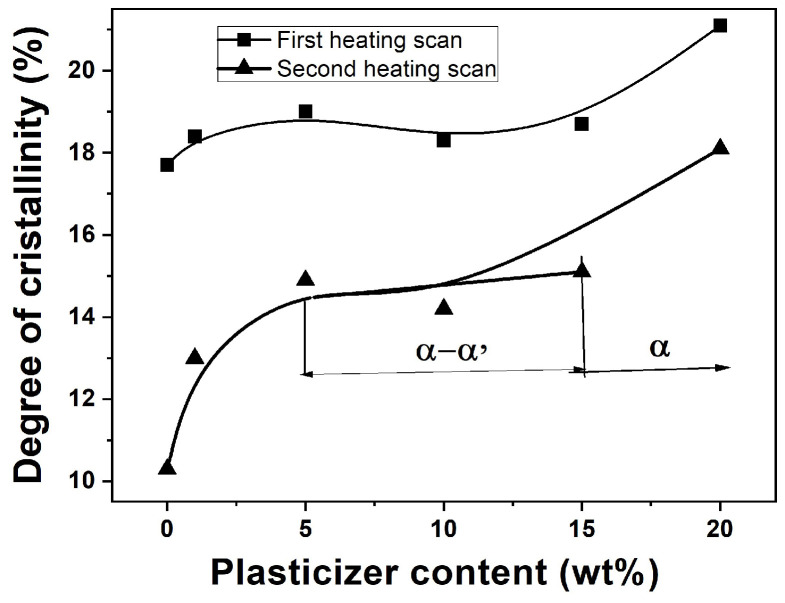
Evolution of the degree of crystallinity (χ_c_) of PLA/PEG blends as a function of PEG content, calculated using Equation (10). Data are derived from the first and second DSC heating scans, corresponding to the thermograms in Figure 1a,b and the data in Table 1 and Table 2.

The evolution of χ_c_ with PEG content, calculated using Equation (10), is illustrated in Figure 3 for both the first and second heating scans (corresponding to Figure 1a,b and Table 1 and Table 2).

During the first heating scan, all samples exhibited a single melting peak and a nearly constant crystallinity degree (χ_c_ ≈ 18–20%) across the entire PEG concentration range (0–20 wt%). This consistently high crystallinity originates from the sample preparation method (solvent casting without thermal pretreatment), which promotes a semi-crystalline structure.

In the second heating scan, three distinct regions were identified based on the PEG concentration:

- Region 1 (0–5 wt% PEG): χ_c_ increased from 10.3% to 14.9%, indicating that even low PEG content enhances chain mobility and facilitates crystallization.
- Region 2 (5–15 wt% PEG): A crystallinity plateau (χ_c_ ≈ 15%) was observed, coinciding with the appearance of double melting peaks. The lower-temperature peak corresponds to the melting of initial α-form crystals, while the higher-temperature peak arises from the disordered α′-form which undergoes melt-recrystallization into the ordered α-form [21,22,29,30,31,32,33,34,35]. The constant χ_c_ in this region suggests a balance between increased nucleation (driven by PEG) and the growing proportion of the α′-form, which has a lower melting enthalpy.
- Region 3 (>15 wt% PEG): χ_c_ increased again, reaching 18.1% at 20 wt% PEG. At this concentration, only a single melting peak—characteristic of the α-form—was detected, implying complete transition to the more stable crystalline structure. This value aligns with the crystallinity plateau observed in the first heating scan. This confirms the role of PEG in promoting α-crystal formation at higher concentrations.

The double melting behavior in plasticized PLA, as reported in prior studies [21,22,29,30,31,32,33,34,35], reflects the complex crystallization dynamics influenced by PEG-induced chain mobility and structural reorganization. These findings underscore the effectiveness of PEG 4000 in modulating both the crystalline morphology and the degree of crystallinity in PLA.

### 3.4. Dynamic Mechanical Behavior: The Critical Role of Thermal History

**Figure 4:**
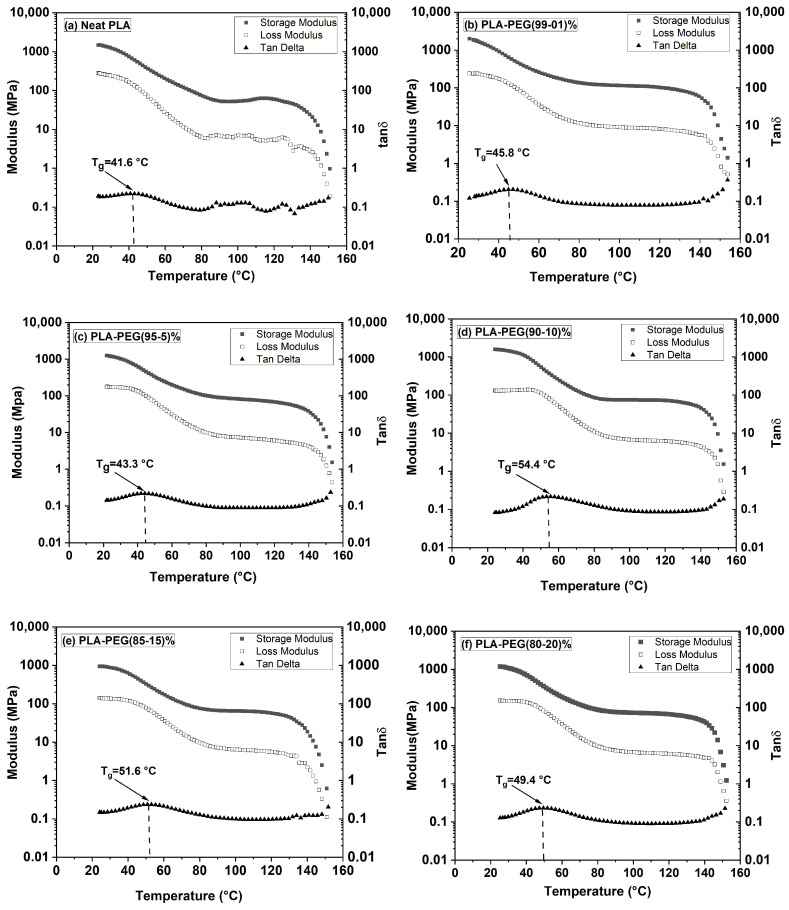
Dynamic mechanical analysis (DMA) of virgin PLA and PLA/PEG blends with their initial thermal history (i.e., without a prior heating–quenching cycle). Temperature dependence of the storage modulus (E′), loss modulus (E″), and loss factor (tan δ) measured at 1 Hz: (**a**) neat PLA, (**b**) 99/1 PLA/PEG, (**c**) 95/5 PLA/PEG, (**d**) 90/10 PLA/PEG, (**e**) 85/15 PLA/PEG, (**f**) 80/20 PLA/PEG.

The dynamic mechanical behavior of polymers, crucial for assessing their performance in flexible packaging, is governed by intermolecular forces and crystallinity. Initial DMA measurements on virgin, solvent-cast PLA/PEG blends (with their inherent thermal history) revealed a minimal modulus drop and an unclear glass transition depression (Figure 4 and Table 3), complicating the assessment of plasticization.

#### 3.4.1. Addressing Thermal History: Experimental Rationale

To test the hypothesis that the initial thermal history masked the plasticizing effect of PEG, a unified thermal protocol was applied prior to dynamic mechanical analysis (DMA), as suggested in the literature to reveal fundamental material properties. All samples were melted at 190 °C for 5 min under a nitrogen atmosphere and then rapidly quenched in ice water to create a predominantly amorphous state with a controlled, erased thermal history. This approach mirrors the common practice in DSC analysis (second heating scan) to reveal intrinsic thermal transitions. While generating a set of controlled semi-crystalline samples via a precise heat–cool–heat cycle (as in DSC) was considered, it was not feasible for DMA due to constraints related to sample dimensions and the extended thermal programming required, which could compromise sample integrity. Therefore, this study focuses on comparing the initial “as-cast” state with the quenched amorphous state to isolate the impact of PEG on chain mobility, independent of the confounding factor of pre-existing crystallinity.

#### 3.4.2. Results on Virgin (As-Cast) Samples

Figure 4 and Table 3 show the storage modulus (E′), loss modulus (E″), and loss factor (tan δ) for virgin PLA/PEG blends at temperatures between 25 and 160 °C. The high initial crystallinity from solvent casting led to a high glassy modulus (E′_g_), a direct transition to a rubbery plateau (E’_N_) without cold crystallization, and broad, flattened tan δ peaks. These results produced inconclusive T_g_ values (46–53 °C) showing no clear dependence on PEG content, consistent with the first DSC heating scan.

#### 3.4.3. Results on Quenched Amorphous Samples: Revealing Plasticization

**Figure 5:**
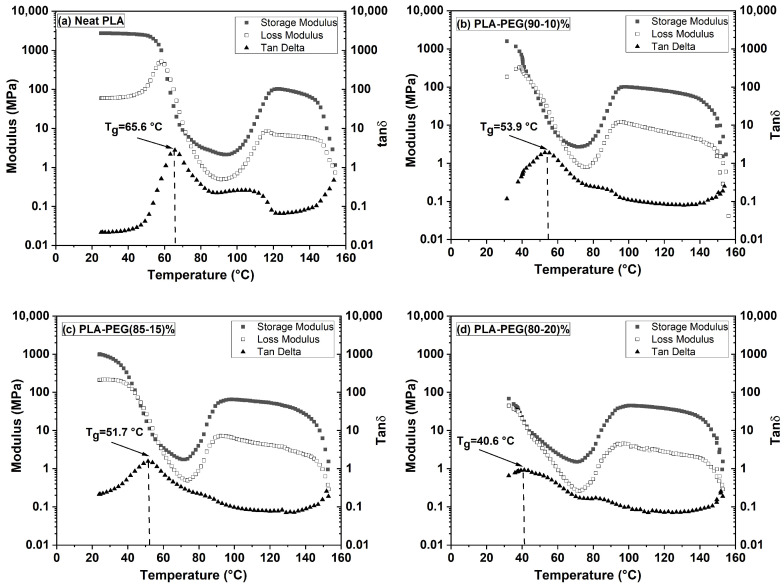
Dynamic mechanical analysis (DMA) of PLA/PEG blends after erasing thermal history (heated to 190 °C and rapidly quenched). Temperature dependence of the storage modulus (E′), loss modulus (E″), and loss factor (tan δ) measured at 1 Hz: (**a**) neat PLA, (**b**) 90/10 PLA/PEG, (**c**) 85/15 PLA/PEG, (**d**) 80/20 PLA/PEG.

The DMA response changed dramatically after erasing the thermal history (Figure 5, Table 4).

The quenched, amorphous samples exhibited a sharp drop in E′ at the glass transition, followed by a distinct cold-crystallization peak (increase in E′), before reaching the rubbery plateau. Crucially, the onset of the E′ drop and the peak of the tan δ curve (T_g_) systematically shifted to lower temperatures with increasing PEG content. For instance, T_g_ decreased from ~66 °C for neat PLA to ~41 °C for the 80/20 blend (Table 4). This clear T_g_ depression, along with a monotonic decrease in the glassy modulus (E′_g_), unequivocally demonstrates the plasticizing effect of PEG when measured in a state free of confounding crystallinity.

#### 3.4.4. Conclusion of the DMA Analysis

This comparative analysis highlights the paramount importance of thermal history in characterizing PLA/PEG blends. The initial, highly crystalline state masked the plasticizing action of PEG. By adopting a unified quenching protocol to create an amorphous reference state, the DMA results clearly reveal the expected enhancement in chain mobility and depression of T_g_ with increasing PEG content. This finding resolves the initial ambiguity and confirms that the observed non-monotonic rheological response observed and discussed in Section 3.5 originates from the molecular-level plasticization effect of PEG which is now clearly evidenced in the amorphous state.

### 3.5. Rheological Properties

#### 3.5.1. Introduction and Experimental Rationale

The melt rheology of polymers is exquisitely sensitive to macromolecular architecture, chain mobility, and the presence of additives. For plasticized systems, rheology provides fundamental insight into how a plasticizer alters chain dynamics and the resulting melt structure. In this study, we observed a non-monotonic variation in complex viscosity for blends containing 5–15 wt% PEG (Section 3.2, DSC). To elucidate the origin of this anomaly—whether it stems from persistent residual crystallinity (α’ precursors) or from unique molecular-level interactions—a targeted rheological investigation was designed.

Following the same rationale applied in DMA (Section 3.4.1), samples were prepared with two distinct thermal histories: (i) with their initial ‘as-cast’ history, and (ii) after erasing thermal history by quenching from 190 °C to create a predominantly amorphous state. For each state, small-amplitude oscillatory shear (SAOS) frequency sweeps were performed at 180 °C and 190 °C. This comparative approach is critical: if the viscosity anomaly disappears at the higher temperature or in the quenched state, it would implicate residual crystallinity. However, if the anomaly persists, this would suggest the formation of a transient network or specific interactions facilitated by PEG4000.

#### 3.5.2. Rheology of Blends with Initial Thermal History

**Figure 6:**
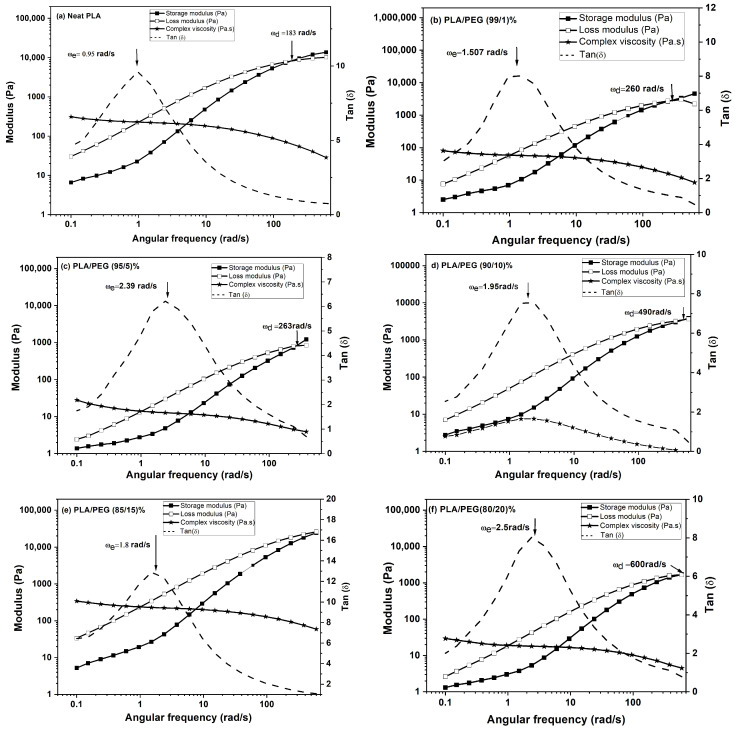
Rheological properties as a function of angular frequency for neat PLA and PLA/PEG blends at 180 °C (with initial thermal history): (**a**) neat PLA, (**b**) 99/1, (**c**) 95/5, (**d**) 90/10, (**e**) 85/15, (**f**) 80/20.

Figure 6 presents the master rheograms (storage modulus G′, loss modulus G″, complex viscosity |η*|, and tan δ) for neat PLA and its blends at 180 °C. All compositions exhibited typical viscoelastic liquid behavior, with G″ > G′ across the measured frequency range and shear-thinning at high frequencies.

A more detailed analysis reveals two characteristic frequencies (Table 5): the entanglement frequency (ωₑ), identified by the maximum in tan δ, and the disentanglement frequency (ω_d_), the crossover point where G′ becomes greater than G″ at high frequencies. Below ω_d_, the material response is more solid-like (G′ > G″). The increase in both ωₑ and ω_d_ with initial PEG addition (up to 5 wt%) indicates enhanced chain mobility due to plasticization.

**Figure 7:**
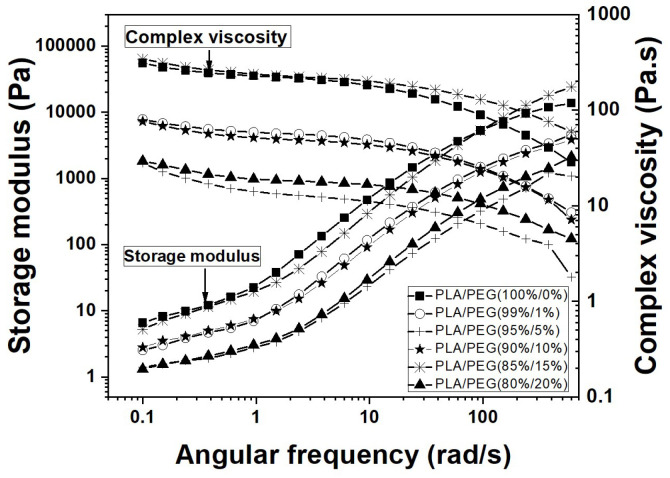
Frequency dependence of complex viscosity (|η*|) and storage modulus (G′) for PLA/PEG blends with initial thermal history at 180 °C.

**Figure 8:**
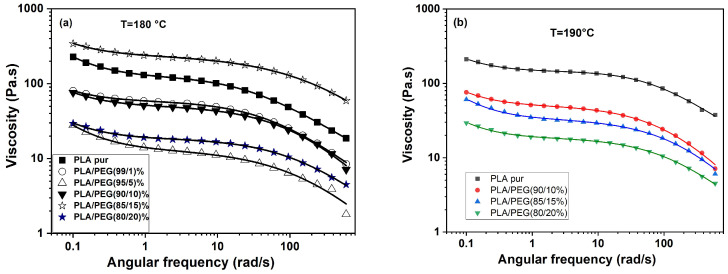
Complex viscosity (|η*|) as a function of angular frequency for PLA/PEG blends with initial thermal history at (**a**) 180 °C and (**b**) 190 °C. Symbols: experimental data; Solid lines: Carreau–Yasuda model fit.

The complex viscosity curves (Figure 7 and Figure 8a) revealed the core anomaly. While |η*| decreased as expected from neat PLA to the 5 wt% PEG blend, it showed a pronounced increase for the 10 and 15 wt% compositions, before decreasing again at 20 wt% PEG.

To quantify this behavior, the data were fitted with the yield-stress Carreau–Yasuda model (Equation (9)). The fitted parameters (Table 6) clearly show the non-monotonic trend: the zero-shear viscosity (η_0_*) drops from 123 Pa·s (neat PLA) to 13 Pa·s (5 wt% PEG), then rises sharply to 241 Pa·s (15 wt% PEG), before falling to 18 Pa·s (20 wt% PEG).

Effect of higher temperature (190 °C): When measured at 190 °C (Figure 8b, Table 7), the viscosity anomaly for the 10–15 wt% blends was significantly attenuated. The η_0_* value for the 15 wt% blend dropped from 241 Pa·s (180 °C) to 33 Pa·s (190 °C). This strong temperature dependence is a first indicator that the anomaly is linked to a thermally labile structure, such as residual or poorly melted crystals.

#### 3.5.3. Rheology of Quenched Blends (Amorphous State)

To definitively eliminate the variable of pre-existing crystalline order, samples were quenched from the melt (190 °C) to create an amorphous state, as done for DMA.

**Figure 9:**
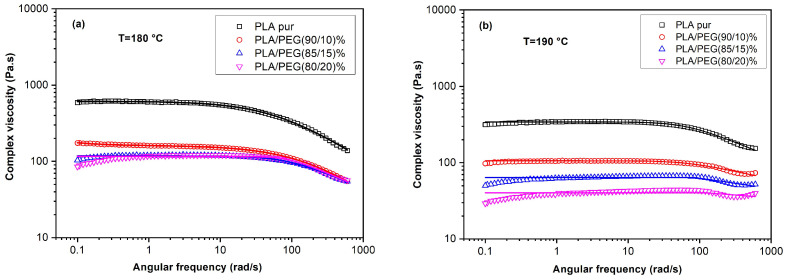
Complex viscosity (|η*|) as a function of angular frequency for PLA/PEG blends after erasing thermal history (quenched) at (**a**) 180 °C and (**b**) 190 °C. Experimental data are represented by symbols, and the solid lines correspond to the fits obtained with the yield stress Carreau–Yasuda model.

The rheological response changed profoundly (Figure 9, Table 8 and Table 9). The pronounced viscosity hump at intermediate PEG concentrations disappeared entirely. The flow curves became simpler and the Carreau–Yasuda model, effective for the “as-cast” samples, could not adequately capture the terminal flow region of the quenched samples, especially at high PEG content and 190 °C (see low R^2^ values in Table 9). This indicates a fundamental change in relaxation dynamics.

Most importantly, the viscosity now decreased monotonically with increasing PEG content at both temperatures, as expected for a conventional plasticizer. The higher absolute viscosities of the quenched samples compared to their “as-cast” counterparts are consistent with their fully amorphous, unrelaxed state.

#### 3.5.4. Synthesis and Interpretation

The combined rheological analysis provides a conclusive answer to the central question posed by the non-monotonic viscosity trend:

- The anomaly (viscosity increase at 10–15 wt% PEG) is strongly suppressed at 190 °C compared to 180 °C.
- The anomaly completely disappears when the samples are analyzed in a quenched, amorphous state with no thermal memory.

These observations unequivocally support the hypothesis that the anomalous rheological response in the “as-cast” blends is primarily due to residual crystallinity. The double-melting behavior observed by DSC (~150 °C) suggests the presence of less-ordered α’ crystals or crystal precursors that are not fully molten at 180 °C. These residual crystalline domains act as physical crosslinks, increasing the melt viscosity and elasticity. At 190 °C or in the quenched amorphous state, these structures are eradicated, revealing the underlying, monotonic plasticizing effect of PEG4000.

This interpretation is consistent with the DMA findings (Section 3.4), where the plasticizing effect (T_g_ depression) was only clearly observable after erasing thermal history. It also aligns with literature suggesting that PEG of higher molecular weight (e.g., 4000 g/mol) can promote different crystallization kinetics and crystal forms in PLA. The single, composition-independent relaxation mode observed in all blends confirms the miscibility of the system and rules out macroscopic phase separation as the cause of the anomaly.

In conclusion, while PEG4000 effectively plasticizes PLA by increasing chain mobility and depressing T_g_, its interaction can lead to a complex crystallization landscape. The resulting residual crystallinity can dominate the melt rheology at temperatures just above the nominal melting point, presenting as a non-monotonic viscosity profile. This study underscores the critical importance of controlling and reporting thermal history when evaluating the properties of plasticized semi-crystalline polymers.

## 4. Conclusions

This study provides a comprehensive analysis of the plasticization of PLA with PEG 4000, elucidating the critical interplay between plasticizer efficiency, thermal history, and crystalline morphology in determining the final material properties. The integrated results from DSC, DMA, and rheology converge on a coherent narrative with significant implications for material design.

First, DSC analysis confirmed the miscibility of PEG 4000 with PLA, at concentrations up to 20 wt%, as validated by the Fox equation. The systematic depression of T_g_ with increasing plasticizer content, reaching a reduction of over 19 °C for the 80/20 blend, unequivocally demonstrates the effectiveness of PEG 4000 in enhancing chain mobility. The crystallinity of the blends evolved in a non-monotonic manner, influenced by the plasticizer-facilitated chain dynamics and the potential coexistence of α′ and α crystalline forms. This is evident from double melting endotherms within certain concentration ranges.

The DMA results delivered a pivotal insight: the thermal history is a dominant factor governing the observed mechanical response. In their initial, solvent-cast state, the high crystallinity of the blends masked the plasticizing effect on the storage modulus and obscured the T_g_. However, after erasing this history via melt-quenching to create a controlled amorphous state, DMA clearly revealed the expected and significant depression of T_g_ and reduction in the glassy modulus with increasing PEG content. This underscores that the true plasticizing efficiency can only be accurately assessed when the confounding variable of pre-existing crystallinity is eliminated.

The most significant mechanistic insight arose from the rheological investigation. An anomalous increase in complex viscosity and elasticity was observed for blends containing 5–15 wt% PEG when measured at 180 °C in their initial state. A targeted experimental strategy—comparing rheology at 180 °C and 190 °C on both “as-cast” and quenched samples—allowed us to decouple the underlying causes. The fact that this anomaly was strongly suppressed at 190 °C and entirely absent in quenched, amorphous samples provides conclusive evidence that it originates from residual crystalline structures (α′ precursors) that are not fully molten at 180 °C, rather than from a transient molecular network. This finding resolves the apparent contradiction between plasticization and increased melt viscosity, highlighting how processing temperature relative to the complex melting landscape dictates the melt structure.

In summary, PEG 4000 is a highly effective plasticizer for PLA, capable of significantly enhancing flexibility and depressing T_g_. Its interaction with PLA, however, creates a nuanced crystallization behavior that can lead to persistent crystalline domains influencing the melt state. This study demonstrates that the thermal history and the precise thermal protocol during testing are not mere experimental details but are central to interpreting and predicting the properties of plasticized PLA. For applications such as extrusion or film blowing, this means that process parameters must be carefully optimized above a critical temperature (≥190 °C) to ensure a homogeneous, fully plasticized melt, thereby unlocking the tailored properties required for advanced biodegradable packaging.

## Figures and Tables

**Table 1:** Thermal properties of neat PLA and PLA/PEG blends obtained from the first heating scan by DSC: melting temperature (T_m_) and melting enthalpy (ΔH_m_). The degree of crystallinity χ_c_ was calculated using Equation (10).

PLA/PEG (wt%/wt%)	T_g_ (°C)	T_cc_ (°C)	ΔH_cc_ (J/g)	T_m_ (°C)	ΔH_m_ (J/g)	χ_c_ (%)
100/00	-	-	-	151.5	16.4	17.7
99/01	-	-	-	150.3	16.7	18.4
95/05	-	-	-	145.5	16.8	19.0
90/10	-	-	-	150.6	15.3	18.3
85/15	-	-	-	152.5	14.8	18.7
80/20	-	-	-	151.7	15.7	21.1

**Table 2:** Thermal properties obtained from the second heating scan by DSC, showing the glass transition (T_g_), cold crystallization (T_cc_), and melting behavior after erasing the thermal history. χ_c_ was calculated using Equation (10).

PLA/PEG (wt%/wt%)	T_g_ (°C)	T_cc_ (°C)	ΔH_cc_ (J/g)	T_m1_ (°C)	T_m2_ (°C)	ΔH_m_ (J/g)	χ_c_ (%)
100/00	59.7	124.3	4.4	-	152.5	9.6	10.3
99/01	56.9	120	8.8	-	151.0	12	13.0
95/05	41.1	89.3	11.2	141.4	148.2	13.2	14.9
90/10	34.9	87.2	6.3	145.3	152.6	11.9	14.2
85/15	29.5	83.8	4.5	146.0	153.4	11.9	15.1
80/20	19.2	77.8	2.9	145.5	153.4	13.5	18.1

**Table 3:** Dynamic mechanical and thermal properties of virgin PLA/PEG blends (initial “as-cast” thermal history).

PLA/PEG (wt/wt)	E’_g_ (MPa)	E’_N_ (MPa)	T_g_ from DMA (°C)	T_m_ (°C)
100/0	1488	52.1	41.6	131.2
99/1	1479	113.8	45.8	136.0
95/5	1212	82.7	43.3	134.1
90/10	1532	74.8	54.4	138.3
85/15	903	64.8	51.6	129.6
80/20	1149	71.8	49.4	140.3

**Table 4:** Dynamic mechanical and thermal properties of PLA/PEG blends after melting at 190 °C and rapid quenching (amorphous state).

PLA/PEG (wt/wt)	E′_g_ (MPa)	E′_N_ (MPa)	T_g_ (°C)	T_m_ (°C)
100/0	2740	100.0	65.6	144.5
90/10	1590	92.6	53.9	135.8
85/15	1006	65.0	51.7	135.0
80/20	70	44.0	40.6	135.0

**Table 5:** Characteristic frequencies for PLA/PEG blends with initial thermal history at 180 °C.

PEG (wt%)	0	1	5	10	15	20
ω_d_ (s^−1^)	183	260	263	290	>600	600
ω_e_ (s^−1^)	0.95	1.51	2.39	1.95	1.80	2.50

**Table 6:** Carreau–Yasuda model parameters for blends with initial thermal history at 180 °C (data from Figure 8a).

PEG (wt%)	σ_0_ (Pa)	η_0_ (Pa·s)	λ (s)	a	n	R^2^
0	10.53	123.37	0.0401	0.9511	0.4132	0.9998
1	2.26	58.25	0.0188	0.8577	0.2646	0.9998
5	1.49	13.11	0.0048	0.6678	4.57 × 10^−14^	0.9984
10	2.65	50.34	0.0072	0.7635	8.57 × 10^−17^	0.9993
15	10.81	241.26	0.0024	0.5719	5.37 × 10^−13^	0.9993
20	1.16	18.39	0.0089	0.8214	0.2641	0.9979

**Table 7:** Carreau–Yasuda model parameters for blends with initial thermal history at 190 °C (data from Figure 8b).

PEG (wt%)	σ_0_ (Pa)	η_0_ (Pa·s)	λ (s)	a	n	R^2^
0	6.89	143.24	0.0164	1.0461	0.4112	0.9992
10	2.65	50.34	0.0072	0.7635	8.59 × 10^−17^	0.9993
15	2.90	33.13	0.0047	0.7332	5.44 × 10^−15^	0.9970
20	1.16	18.39	0.0089	0.8214	0.2641	0.9979

**Table 8:** Carreau–Yasuda model parameters for quenched blends at 180 °C (data from Figure 9a).

PEG (wt%)	σ_0_ (Pa)	η_0_ (Pa·s)	λ (s)	a	n	R^2^
0	0	612.75	0.0157	0.9184	0.3822	0.9988
10	1.46	161.33	0.0062	0.8715	0.3332	0.9996
15	0	117.79	0.0110	1.6037	0.5890	0.9670
20	0	116.28	0.0113	2.8523	0.6036	0.7862

**Table 9:** Carreau–Yasuda model parameters for quenched blends at 190 °C (data from Figure 9b).

PEG (wt%)	σ_0_ (Pa)	η_0_ (Pa·s)	λ (s)	a	n	R^2^
0	0	341.73	0.0158	1.8581	0.6213	0.9811
10	0	105.99	0.0152	1.8239	0.7992	0.9524
15	0	63.95	0.0115	364.24	0.8641	0.3727
20	0	40.43	0.0082	444.19	0.9282	-

## Data Availability

The original contributions presented in this study are included in the article. Further inquiries can be directed to the corresponding author.
